# Transformation to a patient-centred medical home led and delivered by an urban Aboriginal and Torres Strait Islander community, and association with engagement and quality-of-care: quantitative findings from a pilot study

**DOI:** 10.1186/s12913-023-09955-x

**Published:** 2023-09-06

**Authors:** Saira Mathew, Federica Barzi, Anton Clifford-Motopi, Renee Brown (Nunuccal), James Ward (Pitjantjatjara and Nukunu), Richard Mills, Lyle Turner, Antoinette White (Palawa and Iningai), Martie Eaton, Danielle Butler

**Affiliations:** 1 The Institute for Urban Indigenous Health Ltd, 22 Cox Road, Windsor, Qld 4030 Australia; 2 The University of Queensland, Poche Centre for Indigenous Health, 74 High Street, Toowong, Qld 4066 Australia; 3 National Centre for Epidemiology and Population Health, Australian National University, Canberra, ACT 2601 Australia

**Keywords:** Patient centred-medical home, Indigenous health, Care processes, Primary health care, Aboriginal and Torres Strait Islander peoples

## Abstract

**Background:**

The patient-centred medical home (PCMH) is a model of team-based primary care that is patient-centred, coordinated, accessible, and focused on quality and safety. In response to substantial population growth and increasing demand on existing primary care services, the Institute for Urban Indigenous Health (IUIH) developed the IUIH System of Care-2 (ISoC2), based on an international Indigenous-led PCMH. ISoC2 was piloted at an urban Aboriginal and Torres Strait Islander Community-Controlled Health Service in South-East Queensland between 2019–2020, with further adaptations made to ensure its cultural and clinical relevance to local Aboriginal and Torres Strait Islander people. Little is known on the implementation and impact of PCMH in the Australian Indigenous primary care setting. Changes in implementation process measures and outcomes relating to engagement and quality-of-care are described here.

**Methods:**

De-identified routinely collected data extracted from electronic health records for clients regularly attending the service were examined to assess pre-post implementation changes relevant to the study. Process measures included enrolment in PCMH team-based care, and outcome measures included engagement with the health service, continuity-of-care and clinical outcomes.

**Results:**

The number of regular clients within the health service increased from 1,186 pre implementation to 1,606 post implementation; representing a small decrease as a proportion of the services’ catchment population (38.5 to 37.6%). In clients assigned to a care team (60% by end 2020), care was more evenly distributed between providers, with an increased proportion of services provided by the Aboriginal and Torres Strait Islander Health Worker (16–17% versus 10–11%). Post-implementation, 41% of clients had continuity-of-care with their assigned care team, while total, preventive and chronic disease services were comparable pre- and post-implementation. Screening for absolute cardiovascular disease risk improved, although there were no changes in clinical outcomes.

**Conclusions:**

The increase in the number of regular clients assigned to a team and their even distribution of care among care team members provides empirical evidence that the service is transforming to a PCMH. Despite a complex transformation process compounded by the COVID-19 pandemic, levels of service delivery and quality remained relatively stable, with some improvements in risk factor screening.

**Supplementary Information:**

The online version contains supplementary material available at 10.1186/s12913-023-09955-x.

## Background

As an enactment of self-determination, Aboriginal and Torres Strait Islander Community-Controlled Health Services (ACCHS) have led the way in innovative service delivery in Australia [1–4]. Through providing holistic culturally responsive primary healthcare (PHC) services, with an explicit focus on social and cultural determinants of health, the sector has reduced barriers to care all too often experienced by Aboriginal and Torres Strait Islander people [4–7]. Continuous access to, as well as quality and culturally safe care within PHC services is essential for improving health and wellbeing, including the prevention and management of chronic conditions [2, 8]. Evidence from systematic reviews have highlighted that access to PHC prevents progression of chronic diseases and reduces potentially preventable hospitalisations [9, 10].

The Institute for Urban Indigenous Health (IUIH) is a community-controlled regional health organisation that delivers culturally appropriate PHC services across Southeast Queensland (SEQ) for Aboriginal and Torres Strait Islander clients [2]. IUIH supports universal PHC by providing a range of extensive services that result in a “one stop shop” for clients [2]. Relational care – strong and trusting relationships between clients and their healthcare team, with a focus on building connections– is central to culturally responsive high-quality PHC [5] and underpins how IUIH delivers services [2, 11].

Since its establishment in 2009, IUIH has been responsive to community needs in the context of a rapidly growing Aboriginal and Torres Strait Islander population in SEQ. IUIH routinely reviews their clinical care systems, as well as implements continuous quality improvement initiatives and evaluations to ensure quality comprehensive primary care is delivered to their clients [12]. Where required, changes in service delivery are implemented.

The IUIH System of Care Version 2 (ISoC2) is an innovative service reform building on the existing IUIH model of care. This model was implemented in response to the continued rapid growth of the Aboriginal and Torres Strait Islander population in SEQ, and with an intent to recentre the model of care around connections with clients and their families and to foster greater client autonomy. ISoC2 is based on the only published example of a patient-centred medical home (PCMH) developed by, and for, Indigenous peoples [13, 14], with principles closely aligned with IUIH’s ways of delivering primary care services. Further adaptations were made to ensure its cultural and clinical relevance to local Aboriginal and Torres Strait Islander people. PCMHs have proven to be effective for improving the experiences of patients and staff [15], quality of care and health outcomes for patients with complex needs [16].

The defining features of a PCMH include: health care delivered by a team of multidisciplinary clinicians; the voluntary enrolment of clients to the program; a focus on patient education and self-management; the use of technology to support client care (including data-driven improvement), and service planning and co-ordination [17]. ISoC2 represents many of these key features, but with innovation and adaptation to strengthen access, client-provider relationships, and client engagement and agency [12]. This includes assignment to a core multi-disciplinary team, who have an expanded and intersecting scope of practice and work collectively throughout a care episode to meet patient needs. Importantly, each team includes an administrative coordinator and an Aboriginal and Torres Strait Islander health worker (AHW) with a focus on social and cultural wellbeing and client advocacy (see Table 1 and supplementary Table S1).

**Table 1:** Comparison of aboriginal community controlled health services in Australia, patient-centred medical homes and the ISoC2 model of care

In Australia, Aboriginal and Torres Strait Islander Community Controlled services (ACCHS) are locally initiated and operated primary health care services established by the Aboriginal and Torres Strait Islander community. Their purpose is to provide comprehensive, holistic, and culturally responsive healthcare to the Aboriginal and Torres Strait Islander community. Many ACCHS share features of PCMHs including multidisciplinary clinicians available within the service, voluntary enrolment to a specific service, a focus on patient education and self-management, and data-driven continuous quality and service improvement. ISoC2 incorporates additional features including patient-initiated assignment to a core multidisciplinary care team and an explicit focus on relationship-based care. Care team members, particularly non-general practitioner providers, have an expanded and intersecting scope of practice and work collectively throughout a care episode to meet patient needs. Further, data-driven stratification of healthcare resources according to patient needs (cultural, emotional, social and physical) is used to improve care coordination. See supplementary Table S1 for further details.	

Empirical evidence from Australian and international studies has shown that implementation of a PCMH in primary care settings results in improvements in client access to care, increased preventative and follow-up care, and reduction in costs [15, 18]. Other studies have reported an improvement in targeted clinical indicators, improved screening and management of chronic conditions [15, 18, 19]. No studies, however, have assessed these outcomes following implementation of a PCMH specifically in an ACCHS setting. Moreover, few studies have examined the impact of a PCMH on relational continuity-of-care within an Indigenous-led organisation [13, 14]. This study addresses this knowledge gap.

Here we report process, engagement and quality-of-care outcomes collected as part of an evaluation of the ISoC2 pilot program. First, we describe changes in measures relating to transformation to ISoC2 from usual care over the first two years of implementation and second, we examine changes in client access, engagement with services, and quality of care (including continuity-of-care) with a focus on chronic conditions screening and management in the first two-years of implementing ISoC2 compared with a two-year period prior to implementation.

## Methods

### Research team

This study included research team members, clinicians, and service delivery manager/leaders of the ISoC2 working group who are First Nations peoples, (RB, JW, KW, AW) and non-Indigenous peoples (SM, ACM, FB, ME, LT, RM, DB).

### Study population and setting

This research took place on Gubbi Gubbi Country, and we acknowledge their continuing connections to land, sea and community and the ongoing sovereignty of Aboriginal and Torres Strait Islander peoples.

ISoC2 was implemented in the Moreton Aboriginal and Torres Strait Islander Community Controlled Services (MATSICHS) Caboolture clinic, in January 2019. Caboolture is an outer northern suburb of Brisbane with a high proportion of Aboriginal and Torres residents relative to the Australian population. This clinic services around 2000 Aboriginal and Torres Strait Islander people. Within the IUIH network of clinics this service is the first site to have their premises redesigned and workforce reconfigured to support the ISoC2 model of care.

The pilot evaluation in which this study is situated was underpinned by the IUIH’s Cultural Integrity Framework [20] to ensure that Aboriginal worldviews, knowledge, realities and terms of reference were privileged throughout the research process. All IUIH operations have overarching community governance and ownership. This has practical expression through a board of directors that combines community-elected and independent skills-based directors, underpinned by a community accountability framework [2]. The ISoC2 working group oversees this project and includes Aboriginal and Torres Strait Islander and non-Indigenous researchers, clinicians, managers and community liaison officers. This group provides cultural and technical oversight of the project and related subprojects, including with respect to data sovereignty, ensuring that what is measured is meaningful, culturally and clinically.

**Fig. 1:**
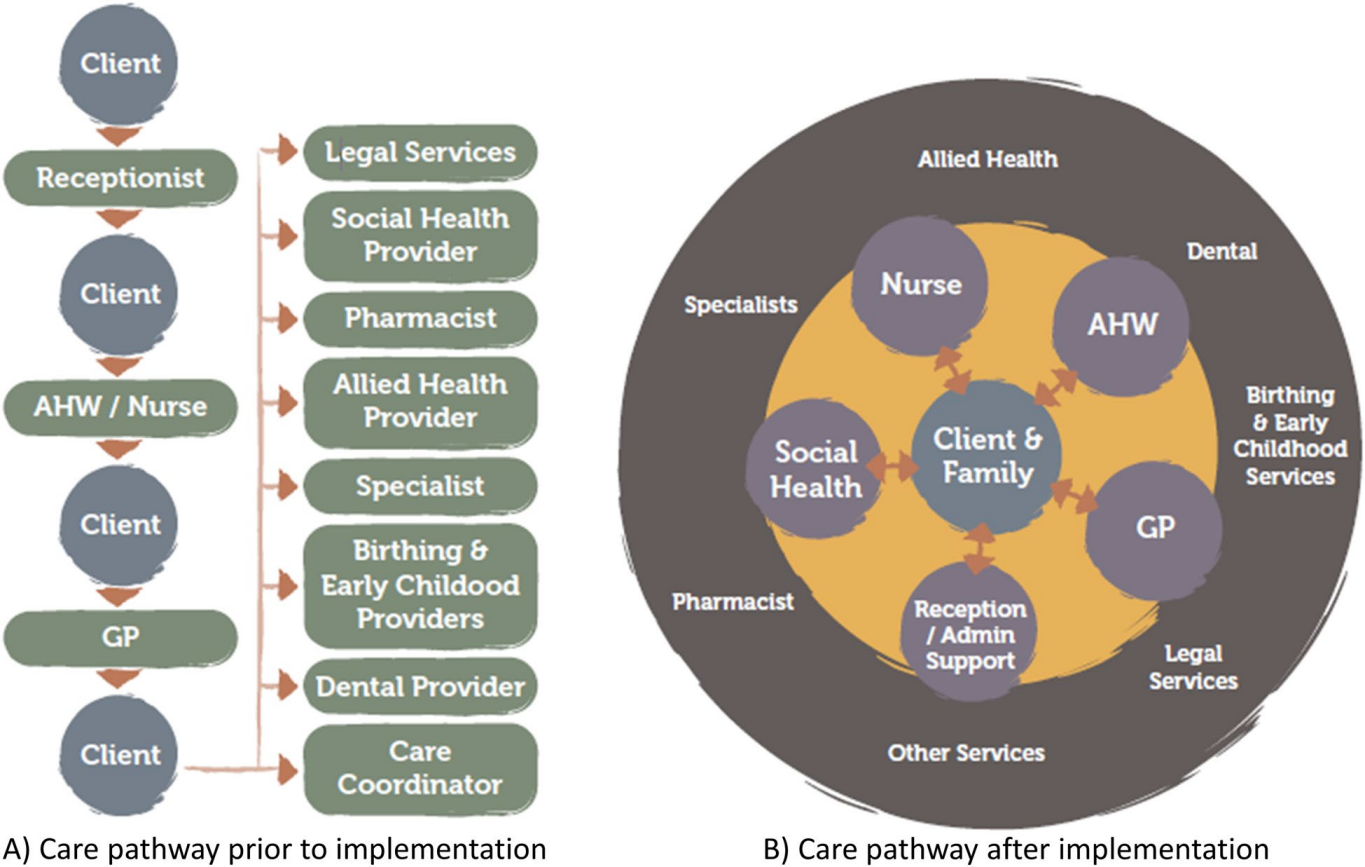
**A** Standard care pathway compared with (**B**) ISoC2 model of care. GP, general practitioner; ISoC2, IUIH System of Care 2

ISoC2 builds on the strengths of the existing IUIH model of care [2] through adaptations intended to: strengthen access, relationship-based care, patient engagement and agency; improve health outcomes; increase efficiency by directing resources within the service to deliver greatest impact; and to scale the service model to cater for growing demand. Patients and their families are assigned to a core multidisciplinary care team – a “Pod”– which includes an Aboriginal or Torres Strait Islander Health Worker (AHW), an administrative coordinator, a registered nurse and a general practitioner (GP, Australia’s primary care physician) working collectively to lead and coordinate care based on the patient’s identified health and wellbeing priorities (Fig. 1 adapted from [12]).

In order to deliver ISoC2 significant clinic restructure and change management occurred within the clinic and among clinicians to enable three teams to deliver the ISoC2 model of care at the Caboolture clinic. Clients could choose a team for their ongoing care. Clients were invited to choose a team when they next attended the clinic after implementation of ISoC2 began. For those clients who did not voluntarily assign to the ISoC2 model, usual care was provided to them.

### Study design

This before and after study aimed to assess engagement and quality of care related to ISoC2. This retrospective quantitative study is part of a larger mixed methods evaluation of ISoC2. Qualitative findings of the evaluation regarding the implementation of ISoC2 from the perspectives of both clients and staff will be reported separately. This study used data extracted from client electronic medical records both prior to ISoC2 implementation 1 January 2016 – December 31 2017 and during the pilot phase 1 January 2019 – 31 December 2020. Data extracted from client records included: socio-demographics, long-term health conditions, medications, clinical measures (e.g. blood pressure, weight, and other investigation results), consultations and Medicare Benefit Schedule (MBS) service item claims (for medical services funded through Medicare, Australia’s universal health insurance scheme).

Our study population were individuals aged 18 years or older, who identified as Aboriginal and/or Torres Strait Islander and were regular clients (defined as at least three visits in the preceding two years [21]) during either the pre-implementation period or the post-implementation period. We excluded those clients who had a death recorded (*n* = 39) during the study period. During 2018, clinic operations were disrupted following a fire at the premises in December 2017. As such, data from this year was excluded.

### Study outcomes

Process measures relating to the implementation of the ISoC2 model and its core components included the number of clients assigned to a care team (as a measure of empanelment) and the proportion of the total numbers of services delivered by each team member (as a measure of the distribution of care between care team members).

Outcome measures related to key objectives of the pilot evaluation included the following:

- *Access* measured as the proportion of regular clients in the total catchment population attending the clinic. The catchment population was based on the estimated resident population within the catchment postcode, adjusted upwards for a 15.4% undercount [22].
- *Engagement and quality of care* including total numbers of services, continuity-of-care score, preventative care (any claim for a preventative health assessment MBS service), chronic disease management among people with a chronic condition (any claim for a chronic disease and complex care management plan or review MBS service), and follow-up care (any claim for a follow-up MBS service among people who have had a health assessment or chronic disease management plan MBS service). The continuity-of-care score was calculated as the proportion of a client’s total visits to the most frequent care team. As per standard methods, at least 4 attendances by an individual in a two-year period were required for calculation, with score of 0.75 is defined as good continuity [23]*.* See supplementary Table S3 for MBS items included in measures using MBS claims data.
- *Recording of behavioural risk factors and clinical outcomes in the electronic health records of clients* relating to screening for behavioural and chronic disease risk factors and monitoring of chronic disease management including recording of smoking status, most recent measured body mass index (BMI), alcohol use; fruit and vegetable consumption, physical activity, measured blood pressure, glycosylated haemoglobin (HbA1c). We also reported the number of cardiovascular (CVD) risk factors recorded that are required to estimate CVD risk using the Framingham risk equation and clinically determined CVD risk. Absolute CVD risk was determined according to current guidelines [24], which combines clinical high-risk criteria and the Framingham risk equation to estimate 5-year absolute risk of a primary CVD event, grouped into low (< 10% risk), moderate (10%–15%) or high (> 15%) absolute risk of a major CVD event in the next 5 years [24].

Study covariates relating to participant characteristics included sociodemographic variables (age, sex) and health characteristics (diagnosis for diabetes, hypertension, renal disease, depression/anxiety, chronic obstructive pulmonary disease/asthma, CVD, number of long-term medications, see supplementary Table S2 for coding health conditions).

### Statistical analyses

Demographic and health characteristics were summarized for the pre and post implementation periods. Age recorded at the beginning of the pre and post implementation period was summarized with mean and standard deviation for each time period. Health conditions diagnosed at any time within each period were all categorical variables and summarized with frequency and percentage, number of comorbidities as a count variable was summarized with median and interquartile range (IQR).

To describe the distribution of care between team members post implementation, the total number of visits with each care team member was computed as the proportion on total visits in one calendar year, separately for clients assigned to a care team and those not assigned. Logistic and linear regression where appropriate was used to quantify age and sex adjusted changes in engagement and quality of care measures over time (pre implementation and post implementation) including the number of visits, and proportion with claims for preventative care and chronic disease management. Given continuity-of-care scores were calculated by care team, this was calculated for the post-implementation period only, and separately for clients assigned to a care team and those not assigned.

Other process and outcome measures were summarized for the pre and post implementation period using age and sex adjusted means for continuous outcomes and age and sex adjusted percentages for categorical variables. Because of the explorative and descriptive nature of this pilot study a formal sample size calculation was not carried out and thus formal statistical testing was not performed throughout the analyses. All analyses were undertaken with the use of a statistical software (Stata, Version 14.2).

## Results

### Participant sociodemographic and health characteristics

There were a total of 1,186 regular clients in the pre implementation period and 1,606 in the post implementation period, with 22% (*n* = 509) of the entire sample in both periods. Participant sociodemographic and health characteristics were similar in both pre and post implementation periods. However, in the post implementation period, the mean age of clients and proportion of women were slightly higher, as was the prevalence of comorbidities and use of polypharmacy (Table 2). In both time periods, more than half of regular clients had depression and/or anxiety (recorded as a diagnosis in the medical record or prescription of a relevant medication).

**Table 2:** Demographic and health characteristics (pre and post implementation)

Characteristic	Pre-implementation (*n* = 1,186)	Post-implementation (*n* = 1,606)
**Age (mean, SD)**	38.6 (14.7)	40.9 (15.8)
**Sex (n, %)**		
Male	487 (41.0)	609 (37.9)
Female	699 (58.9)	997 (62.1)
**Diabetes (n, %)**	229 (19.3)	365 (22.7)
**Hypertension (n, %)**	249 (21.0)	356 (22.2)
**Renal disease (n, %)**	74 (6.2)	119 (7.4)
**Depression/anxiety (n, %)**	624 (52.6)	890 (55.4)
**Asthma (n, %)**	259 (21.8)	347 (21.6)
**CVD (n, %)**	122 (10.2)	161 (10.0)
**No. chronic comorbidities**^**a**^** (median/IQR)**	3 (2 to 4)	3 (2 to 4)
**Comorbidities (n, %)**		
nil	110 (9.2)	163 (10.1)
1–2	461 (38.8)	524 (32.6)
≥ 3	615 (51.8)	919 (57.2)
**No. medications (median[IQR])**	2 [1 to 4]	2 [1 to 5]
**Polypharmacy (n, %)**		
nil	277 (23.3)	316 (19.6)
1–4	644 (54.3)	863 (53.7)
≥ 5	265 (22.3)	427 (26.5)

Includes clients 18 years or older who are regular (those who had more than 3 visits in last two years)

*Abbrev*. *n* number, % Percentage, *IQR* Interquartile range, *CVD* Cardiovascular disease, *SD* Standard deviation

^a^comorbidities includes as separate counts: diabetes, chronic heart disease, ischaemic heart disease, peripheral vascular disease, cerebrovascular disease, hypertension, asthma, chronic obstructive pulmonary disease, chronic lung disease, depression/anxiety, other mental health conditions, behavioural disorders, neurological conditions, obstructive sleep apnoea, thyroid disease, chronic liver disease, chronic kidney disease, dementia, cancer, chronic gastrointestinal conditions, osteoporosis/osteoarthritis. Diagnosis determined from recorded medical history, medications and clinical measures where appropriate. See supplementary Table S1

### Outcomes

#### Process measure relating to implementation of model components

**Fig. 2:**
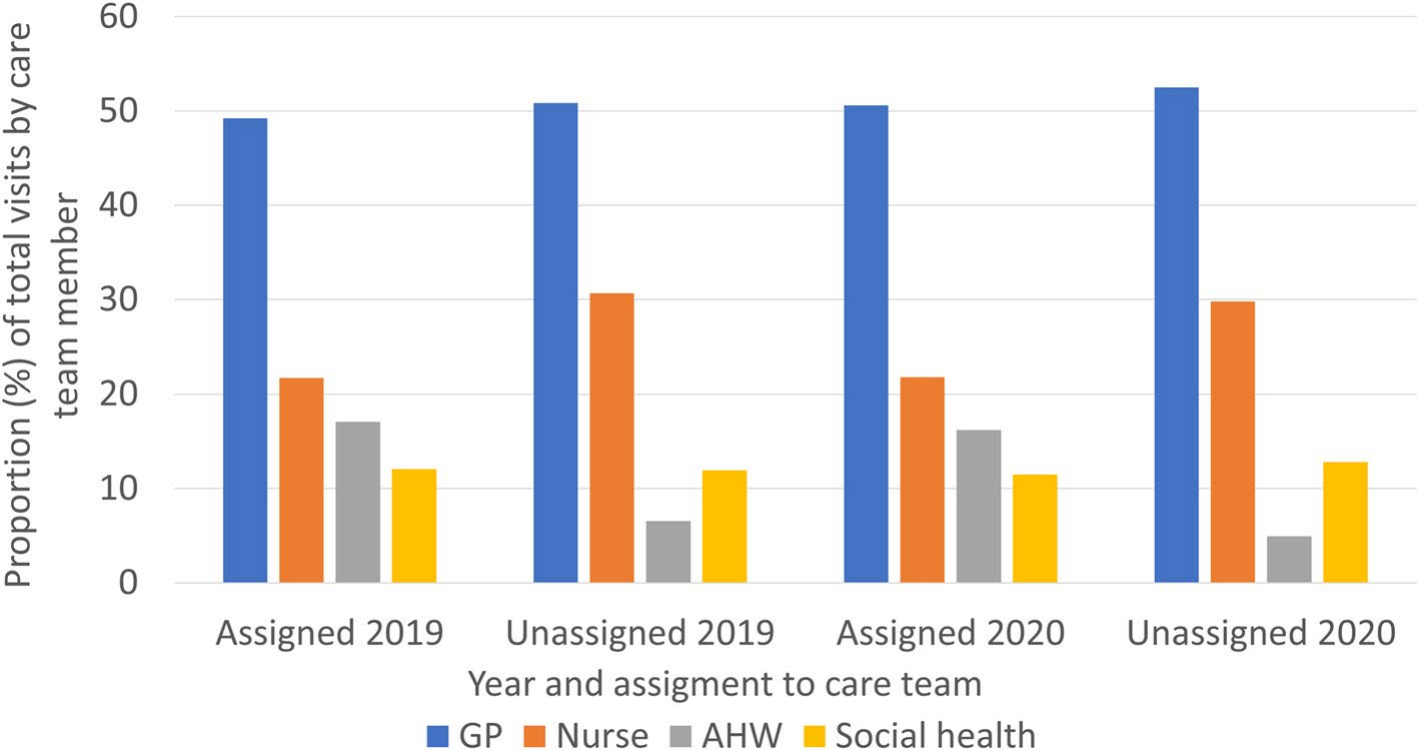
Proportion (%) of visits with each care team member of total visits post-implementation, by year and assignment to a care team. Notes: Includes number of encounters recorded in the electronic health record. Assigned refers to those regular clients who were assigned to a care team during the post-implementation period. Abbrev. GP, general practitioner; AHW, Aboriginal and Torres Strait Islander health worker

By the end of 2020, 58% of regular clients were assigned to a care team with empanelment equally distributed between teams. In the implementation period, for the total client population half of the visits were with the GP (2019 50.1%, 2020 51.6%), a third with the nurse (2019 27%, 2020 26%), 10–11% with the AHW and 12% with the social health worker. When disaggregated by empanelment (Fig. 2), the proportion of visits with the AHW was higher among regular clients assigned to a care team (16–17%).

#### Access

The number of regular clients increased over the study period (Table 2), the percentage of regular clients attending the clinic from the catchment population was largely unchanged (38.6% pre- to 37.7% post-implementation).

#### Engagement and quality-of-care

While there were some fluctuations in the mean number of total visits and claims for preventative care, chronic disease and follow-up services, levels were comparable over the study period, excepting slightly lower levels of chronic disease management and follow up services in 2016 (Table 3). Overall, of clients who had a least 4 services during 2019–2020, 41.8% had continuity-of-care with a care team (42.1% of those assigned to a care team vs 38.8% unassigned).

**Table 3:** Engagement and quality-of-care, number of visits, proportion of regular clients with claims for preventative care and chronic disease management MBS services (95% CI), by year

Service	Mean/proportion (95%CI) 2016	Mean/proportion (95%CI) 2017	Mean/proportion (95%CI) 2019	Mean/proportion (95%CI) 2020
Total number of visits (mean)	17.1 (16.3, 18.0)	17.3 (16.4, 18.1)	15.3 (14.6, 16.1)	16.7 (15.9, 17.5)
Health assessment (%)		85.0 (83.0, 87.1)		87.2 (85.5, 88.8)
Chronic disease and complex care management plan (%)	33.3 (30.6, 36.0)	40.6 (37.8, 43.5)	35.8 (33.4, 38.2)	37.0 (34.6, 39.4)
Chronic disease and complex care management plan review (%)	27.5 (25.1, 29.8)	40.4 (37.8, 43.0)	34.0 (31.8, 36.2)	36.4 (34.2, 38.6)
Follow-up care (%)	49.3 (46.5, 52.1)	52.4 (49.6, 55.2)	57.9 (55.5, 60.3)	54.8 (52.3, 57.2)

Models adjusted for mean age and sex. Proportion who had health assessment calculated over a two-year period pre- and post-implementation. Only those with at least one chronic condition included for estimated proportion with a claim for a chronic disease and complex care management plan, and only those who received a chronic disease management plan included for estimated proportion with a chronic disease management plan review and follow-up service. Follow-up services include MBS claims for allied health, AHW and practice nurse services relating to chronic disease management

*Abbrev*. *AHW* Aboriginal and Torres Strait Islander Health Worker, *CI* confidence interval, *MBS* Medicare Benefits Schedule

#### Recording of behavioural risk factors and clinical outcomes

Recording of smoking status, measured blood pressure and HbA1c levels was unchanged or slightly lower post compared to pre implementation (Table 4). More participants post implementation had sufficient risk factors recorded to calculate absolute CVD risk (pre vs post 26.1% vs 50.7%, according to current guidelines). In terms of levels of risk factors and clinical outcomes, post implementation a lower proportion were currently smoking (52.1 vs 41.9), while mean measured blood pressure among clients with a relevant chronic condition, HbA1c levels among people with diabetes and proportion at high absolute CVD risk were unchanged. Similar patterns for blood pressure and HbA1c were also observed for all regular clients (supplementary Table S4).

**Table 4:** Recording of behavioural risk factors and clinical outcomes for risk factors and chronic disease

Risk factors and clinical outcomes	Pre-implementation (*n* = 1,186)		Post-implementation (*n* = 1,606)	
	**n**	**mean/%(95%CI)**	**n**	**mean/%(95%CI)**
**BMI kg/m2 (mean,95%CI)**		30.4 (30.0–30.7)		30.8 (30.4–31.1)
**BMI (n, %[95%CI])**				
Underweight (≤ 18.5 kg/m^2^)	30	3.1 (2.1–4.1)	45	2.8 (2–3.6)
Normal weight (18.5–25.9 kg/m^2^)	257	24.3 (21.8–26.9)	313	20.9 (18.9–23)
Overweight (25–29.9 kg/m^2^)	280	26.1 (23.5–28.7)	361	24.4 (22.2–26.6)
Obese (≥ 30 kg/m^2^)	498	46.5 (43.6–49.4)	767	51.8 (49.3–54.3)
Missing	121	-	120	-
**Smoking recorded (n, %[95%CI])**	1,165	98.1 (97.3–98.9)	1,561	97.1 (96.3–98.0)
**Smoking status (n, %[95%CI])**				
Current smoker	607	52.1 (49.3–54.9)	654	41.9 (39.4–44.3)
Ex-smoker	260	22.2 (19.9–24.5)	437	28.1 (25.9–30.3)
Non-smoker	298	25.6 (23.1–28.2)	470	30.0 (27.8–32.3)
Missing	21	-	45	-
**BP recorded (n, %[95%CI])**	611	85.4 (82.8–88.0)	986	85.4 (83.3–87.5)
**No. BP measures (mean95%CI])**^***a***^		9.3 (8.5–10.2		5.1 (4.4–5.8)
**Systolic BP mmHg (mean,95%CI)**		128 (127–129)	129	129 (128–130)
**HbA1c recorded (n, %[95%CI])**	178	77.2 (71.8–82.6)	302	82.8 (78.9–86.7)
**No. HbA1c measures (mean, 95%CI)**^**a**^		2.2 (2.0–2.3)		2.1 (2.0–2.2)
**HbA1c % (mean, 95%CI)**		7.3 (7.1–7.6)		7.6 (7.4–7.8)
**Sufficient data to calculate FRE (n, %[95%CI])**	649	54.2 (51.6–56.9)	1062	66.4 (64.2–68.7)
**Sufficient data to calculate ACVR-FRE and clinically determined (n, %[95%CI])**^***b***^	313	26.1 (23.7–28.4)	813	50.7 (48.4–53.1)
**Absolute CVD risk (n, %[95%CI])**^***c***^				
Low risk	422	64.1 (61.3–66.9)	706	63.1 (60.8–65.4)
Moderate risk	24	3.3 (2.0–4.5)	49	4.7 (3.4–6.0)
High risk	236	32.6 (29.8–35.5)	339	32.2 (29.9–34.5)
missing	504	-	512	-

Models adjusted for mean age and sex. Denominator for blood pressure includes regular clients with a diagnosis of hypertension, renal disease, diabetes, prior CVD and at absolute high risk of CVD (pre-implementation *n *= 715, post *n *= 1152); for HbA1c includes regular clients with a diagnosis of diabetes (pre-implementation *n *= 250, post *n *= 394). Mean BP and HbA1c based on readings in the previous 12 months

*Abbrev*. *BP* Blood pressure, *BMI* Body mass index, *CVD* Cardiovascular disease, *FRE*, Framingham risk equation, *n* number, % Percentage, *IQR* Interquartile range, *SD* standard deviation

^a^Number of blood pressure and HbA1c measurements in the last 12 months when at least one measure taken

^b^Risk factors for CVD calculation include systolic blood pressure, HDL, Cholesterol, smoking status, gender, and age

^c^includes both clinically determined and Framingham risk equations

## Discussion

This is the first study to empirically examine the impact of a whole-of-practice transformation to a PCMH in an ACCHS setting. In this study, transformation of the service to a PCMH is evident. By the second year of implementation more than a half of regular clients were assigned to a care team, with a greater proportion of their visits with the AHW compared to clients who were not assigned to a care team. Use of services, including chronic disease care planning, follow up services and preventative health assessments, were largely unchanged over the study period, while screening for absolute CVD risk improved. These findings are generally consistent with those from the recent evaluation of the Health Care Homes (HCH) trial in Australia, where patients with chronic and complex health conditions were enrolled in a model of primary care based on the PCMH. Patients enrolled in the HCH had improved care planning, improved access to GPs and more frequent tests for blood pressure and HbA1c, compared to those with standard care [25]. The early findings from the study reported here are encouraging given they occurred in a setting undergoing a significant change process coupled with the emergence of the COVID pandemic in Australia in March of 2020. Aboriginal and Torres Strait Islander people experience high rates of health-related risk factors for severe COVID disease, which are in turn are strongly associated with underlying social and historical determinants of health [26]. As such, widescale transition from in-person consultations to telehealth was imperative. Even so, service levels in the first year of the pandemic were maintained and contrasts with the marked reduction in preventative and chronic disease care observed nationally [27, 28] and internationally [29], as well as overall health service utilisation internationally [29]. This suggests that the ISoC2 model contributed to supporting the delivery of these services.

Clients assigned to a care team had a greater opportunity to see clinicians relevant to their needs with a greater proportion of their visits occurring with an AHW. This is encouraging given the integral role of the AHW in Aboriginal and Torres Strait Islander people’s health and wellbeing [30, 31]. Prior literature has shown that having Aboriginal and Torres Strait Islander people in the health workforce plays a vital role in addressing risk to Indigenous communities and is found to improve health outcomes by providing culturally relevant information and culturally safe forms of care [4]. The ISoC2 model likely increased contact between AHW and clients by creating a relational and dynamic care pathway responsive to the values, needs, and desires of clients, and shared responsibility of clients through the delivery of team-based care [32].

The findings suggests that clients assigned to a Pod were more likely to have continuity-of-care. This is essential for building relationships and client-team partnership, thereby supporting shared decision making and ongoing engagement with services. Sustained engagement with health care is important for the management of chronic conditions and overall wellbeing [33–36]. Further, people who receive regular care from the same providers are more likely to receive better preventive health care and have better health outcomes [33–35].

Findings in relation to screening and monitoring for health-related risk factors and chronic disease were variable. The number of blood pressure measurements decreased while recording of CVD risk factor required to determine absolute risk improved. However, clinical outcomes remained unchanged. This is likely due to being in the early stages of implementation, as well as pandemic-related disruptions to physical measurements in 2020. Previous studies also indicate it requires at least 3–5 years from commencement of transformation to a PCMH to observe changes in clinical outcomes [15]. Screening is key for early detection and management of chronic disease, and it is expected that as transformation to a PCMH progresses this will improve as well as the related clinical outcomes.

### Strengths and limitations

A strength of this study is that it is Indigenous-led and designed, informed by community needs and priorities. Secondly, this is the first Australian study to examine the effectiveness of a whole-of-practice transformation to a PCMH in an ACCHS. In the recently evaluated Australian HCH trials [25], while some ACCHS were involved, for most practices only a limited number of patients from each site were enrolled in this model of care impacting the implementation and hence effectiveness of these models. Further, participating ACCHS were solely from rural and remote settings. The current study provides insight into PCMH transformation in the urban ACCHS context. Third, the inclusion of consecutive visits by regular clients, with a relatively low rate of missing assessments on most outcomes, reduced the risk of sampling bias. Limitations that warrant consideration include that the high rates of missing data in the study population for CVD risk may bias estimates. However, the proportion at high absolute CVD risk was comparable to estimates from nationally representative data [37]. The study was limited to a single intervention site without comparison standard care data, hence findings may not be generalisable and there is less certainty that changes are attributable to the intervention rather than other factors. Given the pilot and preliminary nature of the study, comparison to standard care sites was beyond the scope of the present study. Leveraging from findings of this pilot study, a further study is underway to expand the implementation of the PCMH model to a second site within the IUIH network and compare outcomes to standard care clinics over a five-year period [12]. There were limited data available on important cultural, social and economic determinants of health and wellbeing. To address this gap our subsequent study will include data linkage to self-reported data collected through the Mayi Kuwayi National study of Aboriginal and Torres Strait Islander Wellbeing survey. This survey includes information on socio-cultural determinants of health as well as health and wellbeing outcomes not routinely captured through the health record data [38].

## Conclusions

This study demonstrated that with the implementation of a PCMH in an urban ACCHS, there was improved contact with the AHW and overall continuity-of-care. Moreover, service delivery has been stable, including preventative and chronic disease care. Globally, the pandemic had affected the way services were delivered and the level of engagement from clients, yet this was not apparent to the same extent in this study. ISoC2 has been implemented at additional sites within the IUIH network, and subsequent studies will determine if findings are similar across these different settings. Further, the qualitative component of this study will explore the experiences of both staff and clients during implementation, providing important contextual information for the current findings. The ISoC2 model comprised core components that are aligned with the ACCHS sector and flexible enough to be adapted to the needs and preferences of staff and clients in individual ACCHS settings. For other ACCHS similarly implementing a PCMH in their setting, this study demonstrates that such models are feasible to implement and have the potential to support access to culturally safe care, continuity-of-care, and contribute to changes in preventative health screening activities. PCMH transformation is in its early stages of implementation, and hence marked changes in processes of care and clinical outcomes are unlikely. Our subsequent study will provide a more comprehensive understanding of this model of care and draw more definitive implications on processes of care and clinical outcomes, including the relationship with social and cultural determinants of health for Aboriginal and Torres Strait Islander people. Avenues for future research include exploring to what extent models of care like ISoC2 can respond to workforce challenges, such as part-time work, and maintain the relational care that is central to its design.

### Supplementary Information

**Additional file 1:** **Supplementary Table S1.** Comparison of care components for models of care taken from Butler et al, 2022 [12]. **Supplementary Table S2.** Clinical indicators and case definitions for chronic diseases. **Supplementary Table S3.** Medicare Schedule Benefit (MBS) items included for health assessments and chronic disease services. **Supplementary Table S4.** Processes of care and clinical outcomes for risk factors and chronic disease, total regular client population. 

## Data Availability

The data that support the findings of this study are available from the Institute for Urban Indigenous Health, but restrictions apply to the availability of these data, which were used under license for the current study, and so are not publicly available. Data are however available from the authors upon reasonable request and with permission of the Institute for Urban Indigenous Health.

## Abbreviations

IUIH: Institute for Urban Indigenous Health
PCMH: Patient-centred medical home
ISoC2: IUIH system of care version 2
GP: General practitioner
AHW: Aboriginal and Torres Strait Islander Health Worker
RN: Registered nurse
CVD: Cardiovascular disease
N: Number
%: Percentage
SD: Standard deviation
IQR: Interquartile range
CI: Confidence intervals
BMI: Body mass index
BP: Blood pressure
mmHg: Millimetres of mercury
HbA1c: Haemoglobin A1C
FRE: Framingham risk equation
