# Supplementary material for: Transformation to a patient-centred medical home led and delivered by an urban Aboriginal and Torres Strait Islander community, and association with engagement and quality-of-care: quantitative findings from a pilot study
Source: BMC Health Serv Res. 2023 Sep 6;23:959. doi: 10.1186/s12913-023-09955-x (PMC10483750; doi:10.1186/s12913-023-09955-x)
Supplement: Supplementary file 1 — Additional file 1: Supplementary Table S1. Comparison of care components for models of care taken from Butler et al, 2022 [12]. Supplementary Table S2. Clinical indicators and case definitions for chronic diseases. Supplementary Table S3. Medicare Schedule Benefit (MBS) items included for health assessments and chronic disease services. Supplementary Table S4. Processes of care and clinical outcomes for risk factors and chronic disease, total regular client population. [file 12913_2023_9955_MOESM1_ESM.pdf]

Supplementary files: Transformation to a patient-centred medical home led and delivered by an urban Aboriginal and Torres Strait Islander community, and association with engagement and quality-of-care: quantitative findings from a pilot study.

Supplementary table S1. Comparison of care components for models of care taken from Butler et al, 2022 (1).

Care components	PCMH(2, 3)	IUIH Standard care	ISoC2
Leadership[1]	Leaders fully engaged with the process of change at all levels of the organisation	Community governance and accountability structure	Community governance and accountability structure<br>Distinct operational working group to support model transformation
Patient Enrolment[2]	Assigned to a clinic or 'teamlet' of PCP/PCP assistants	Administration staff or patients assign to preferred GP provider	Voluntary patient-initiated enrolment with a core multidisciplinary care team, a "Pod"
Team based care	Provider working with a team of other providers; may have 2-3 PCP/PCP assistants in a 'teamlet'	Provider working together with teams but work independently<br><br>Care planning scheduled intermittently	Pod members working collaboratively<br><br>Care planning throughout patient journey
Care pathways[3]	Various, in Australia mostly from GP to other services	First contact with administration staff and then to RN/AHW, followed by the GP. GP then refers to other allied or specialist services	Dynamic pathway where Pod members work collaboratively to customise a pathway to meet patient needs
Scope of practice	Various, specific and expanded roles	Traditional discipline and specific roles	Expanded, intersecting scope of practice particularly of non-GP provider.
Relationship-based care and continuity of care	Primarily between PCP/'teamlet' and patient<br>Supports shared decision making	Primarily between GP and patient<br><br>Usually supports shared decision making	Patient and Pod<br><br>Routine use of goal setting and patient-led decision making tools
Use of technology for data-driven care coordination and quality improvement	Shared electronic health record<br>Variable use of data for quality improvement[4]	Shared electronic health record.<br>Data-driven continuous quality improvement in care	Shared electronic health record<br>Data-driven continuous quality improvement in care<br>Data-driven stratification of healthcare resources according to patient needs (cultural, emotional, social and physical)
Access and availability	Use of multiple modalities with extended hours	Use of multiple modalities but mostly face to face	Use of multiple modalities: face to face, telephone and home visits with extended hours
Funding sources[5]	Multiple often blended payments	Blended payments	Blended payments

Notes:

1. ACCHS have a specific governance structure. Community governance and ownership of IUIH has practical expression through a board of directors that combines community-elected and independent skills-based directors, underpinned by a community accountability framework, centred on the principle that decision-making should occur at the closest level possible to clients, families and communities. The operational working group overseeing ISoC2 includes clinicians and managers from participating sites, personnel responsible for workforce development and service implementation, and research and evaluation partners. 2. In ISoC2, a ‘pod’ comprises an administrative coordinator, AHW, RN and GP working together throughout the patient’s care journey. 3. In most circumstances in Australia, including in Health Care Homes(4) (the PCMH implemented in some services in Australia over the last 5 years), most patients will see a GP prior to other providers. 4. In the PCMH model panel registry typically used to manage and improve care. 5. PHC in Australia is funded predominantly through fee-for-service, while PCMH models often have a blended payment (capitation, pay for performance and fee-for-service), while ACCHS have blended payment as the standard funding model. Abbrev. AHW, Aboriginal and Torres Strait Islander health worker; GP, general practitioner; IUIH, Institute for Urban Indigenous Health; PCMH, patient-centred medical home; PCP, primary care physician; RN, registered nurse.

Supplementary table S2. Clinical indicators and case definitions for chronic diseases

Chronic diseases	Clinical indicator/ source data for case definition	Care plan	Observations	Medications (MIMS classification)	Medications	Medical History (Free text)
Type 2 Diabetes	Care plan, medical history, medications, HbA1c≥6.5% on 2 occasions, fasting blood glucose ≥7 on 2 occasions, random blood glucose ≥11 on 2 occasions	Diabetes care plan; Diabetes retinopathy	Fasting blood sugar; random blood sugar; HbA1c	6D, 6E	Insulin & hypoglycaemics	Diabetes; Adult-Onset Diabetes Mellitus; Diabetes Mellitus; non-insulin-dependent Diabetes mellitus; Type 2 diabetes
Chronic Heart disease	Care plan and medical history	Structural heart disease; heart failure	NA	NA	NA	Heart Failure; Ventricular failure; atrial fibrillation; cardiomyopathy, endocarditis; cardiac arrest; chronic heart disease, valvular disorders
Ischaemic Heart disease	Care plan and medical history	Ischaemic Heart Disease	NA	NA	NA	Acute myocardial infarction; heart attack, angina; ischaemic heart disease; coronary heart disease; Atherosclerosis, CABG
Cerebrovascular disease	Care plan and medical history	Cerebrovascular disease	NA	NA	NA	stroke, transient ischaemic attack, cerebrovascular disease, cerebrovascular attack, transient ischaemic event
Hypertension	Care plan and medical history	Hypertension	NA	NA	Agents not specific for hypertension, category	Hypertension; high blood pressure, elevated blood pressure

					included for secondary prevention but not used for case definition	
Asthma	Care plan, medical history, and medications (asthma meds prescribed in last 12 months)	Asthma	NA	11B to 11E	Bronchodilator aerosols and inhalations; preventive aerosols and inhalations	Asthma
Chronic Obstructive Pulmonary Disease	Care plan, medical history, and medications; smoking history and if on bronchodilator	COPD	NA	11B to 11E	Preventive aerosols and inhalations	Chronic obstructive airways disease; chronic obstructive pulmonary disease
Chronic Lung disease	Care plan and medical history	Chronic lung disease	NA	NA	NA	Lung disease -silicosis; pneumococcus; interstitial lung disease; restrictive lung disease; farmers lung
Depression/ Anxiety	Care plan, medical history, and medications	Mental illness	NA	3D	antidepressant s	Depression, major depression, anxiety, generalised anxiety disorder, depressed; obsessive compulsive disorder
Other mental health conditions	Care plan, medical history, and medications	Mental illness	NA	3C	antipsychotics	schizophrenia; psychosis; personality disorder, post traumatic stress disorder, mental illness (NOS)
Behavioural conditions	Medical history	Nil	NA	NA	NA	Autism; Asperger; attention deficit hyperactivity disorder, behaviour

Neurological Conditions	Care plan, medical history, medications	Epilepsy	NA	3E to 3H	Central nervous system agents; anticonvulsants	Epilepsy; migraine; headache disorders
Dementia	Medical history	NA	NA	NA	NA	Dementia; short term memory loss; cognitive impairment
Cancer	Medical history	Nil	NA	NA	NA	breast/colon/colorectal/prostate/ovarian cancer any cancer, melanoma
Chronic gastrointestinal conditions	Care plan and medical history	Nil	NA	NA	NA	Gastro-oesophageal reflux disease; inflammatory bowel disease; peptic ulcer disease; irritable bowel syndrome, gastritis, pancreatitis, bowel obstruction
Osteoporosis/ Osteoarthritis	Care plan, medical history, and medications	Osteoarthritis- Knee and Hip; Osteoporosis; Prolia (Denosumab)	NA	6G, 5B & 5E	Agents affecting calcium and bone metabolism; Antirheumatoid agents	Osteoporosis, osteoarthritis, Prolia (Denosumab)
Thyroid disease	Care plan, medical history, and medications	Thyroid disease	NA	6F	thyroid hormones and antithyroid medications;	Hyperthyroidism; hypothyroidism; Hashimoto’s disease; graves’ disease
Chronic Kidney disease	Care plan, medical history, and medications; observations eGFR stage 3 or 4 or 5, macroalbuminu	Chronic Kidney disease- Stage 3 to 5; Macroalbuminuria – ACR; Renal transplant	eGFR stage 3 or more; ACR macroalbuminuria	NA	NA	Chronic kidney disease; kidney disease; renal impairment; renal failure

	ria based on ACR					
Chronic Liver disease	Care plan and medical history	Chronic Hepatitis B & C; Chronic liver disease	NA	NA	NA	Hepatitis; liver disease; liver failure; cirrhosis; hepatic steatosis
Obstructive sleep apnoea	Care plan and medical history	OSA	NA	NA	NA	sleep apnoea; continuous positive airway pressure

Notes: Abbrev. ACR, albumin-creatinine ratio; COPD, chronic obstructive pulmonary disease; CABG, coronary artery bypass graft; eGFR estimated glomerular filtration rate; HbA1c, glycosylated haemoglobin; OSA, obstructive sleep apnoea. Case definitions based on: whether the condition was listed as a care plan; medical history diagnosis; a prescription related to that condition had been added to a patient record; or clinical observations consistent with a diagnosis of the condition was recorded in the patient record (depending on the condition). Care plans are a standardised template of scheduled activities relating to a particular condition that can be added to the patient record. Medications were categorised using the Monthly Index of Medical Specialties (MIMS) classification of medications (https://www.mims.com.au/index.php)

Supplementary table S3. Medicare Schedule Benefit (MBS) items included for health assessments and chronic disease services.

MBS service category	MBS Item numbers
Health assessments	701-715, 224-228, 92004-92016; 92011-92023
CDM/TCA	721-723, 731, 229-230, 92024, 92068, 92025, 92069, 92055, 92099, 92056, 92100
CDM/TCA reviews	732, 233, 92028, 92072, 92059, 92103
CDM follow-up services	10951-10970, 81305-81360, 93000, 93048, 93013, 93061, 10983-10987, 10987, 10997, 10950, 81300, 93200, 93201, 93202, 93203

Notes: CDM, chronic disease management, TCA; team care arrangements. Phone and video MBS items introduced for whole of population telehealth during the COVID-19 pandemic also included.

Supplementary table S4. Processes of care and clinical outcomes for risk factors and chronic disease, total regular client population

Risk factors and clinical outcomes	Pre-implementation (n=1,186)		Post-implementation (n=1,606)	
	n	mean/%(95%CI)	n	mean/%(95%CI)
**BP recorded (n, %)***	921	77.6(75.3-80.0)	1,275	79.1(77.1-81.2)
**No. BP measures (mean, 95%CI)[a]**		7.5(6.9-8.1)		4.5(4.0-5.0)
**Systolic BP mmHg (mean, 95%CI)**		126(125-127)		127(126-128)
**HbA1c recorded, n (%[95%CI])***	455	38.3(35.6-40.9)	852	52.7(50.3-55.0)
**No. HbA1c measures (mean, 95%CI)[a]**		1.5(1.4-1.5)		1.5(1.4-1.5)
**HbA1c (mean, 95%CI)**		5.8(5.7-5.9)		6.0(5.9-6.0)

Notes: Models adjusted for mean age and sex. [a]Number of blood pressure and HbA1c measurements in the last 12 months when at least one measure taken. [b] Risk factors for CVD calculation include systolic blood pressure, HDL, Cholesterol, smoking status, gender, and age; [c] includes both clinically determined and Framingham risk equations.

References

1. Butler D, Clifford-Motopi A, Mathew S, Nelson C, Brown R, Gardner K, et al. Study protocol: primary healthcare transformation through patient-centred medical homes—improving access, relational care and outcomes in an urban Aboriginal and Torres Strait Islander population, a mixed methods prospective cohort study. BMJ Open. 2022;12(9):e061037.

2. Bodenheimer T, Ghorob A, Willard-Grace R, Grumbach K. The 10 Building Blocks of High-Performing Primary Care. The Annals of Family Medicine. 2014;12(2):166-71.

3. Primary Care Collaborative. Defining the medical home. A patient-centered philosophy that drives primary care excellence [18/07/2022]. Available from: https://www.pcpcc.org/about/medical-home.

4. Health Policy Analysis. Evaluation of the Health Care Homes program-interim evaluation report 2020, Volume 1: Summary report. Canberra: Department of health; 2020.
